# Using Functional Resonance Analysis Method (FRAM) Modelling to Assess the Factors That Slow or Prevent Clinicians in Performing Advanced Life Support (ALS) During Crash Calls to Park House Mental Health Hospital

**DOI:** 10.7759/cureus.82231

**Published:** 2025-04-14

**Authors:** Benjamin R Smyth, Swanand Patwardhan, Eve L Turner, Sian McLintock

**Affiliations:** 1 General Internal Medicine, Manchester University NHS Foundation Trust, Manchester, GBR; 2 Psychiatry, Greater Manchester Mental Health NHS Foundation Trust, Manchester, GBR

**Keywords:** advanced life support, analysis methodology, cardiac arrest team performance, physical health in psychiatry, quality improvement tool

## Abstract

This Quality Improvement Project (QIP) aimed to improve the response system for crash calls at Park House Mental Health Hospital, supported by the North Manchester General Hospital Crash Team. Using a functional resonance analysis method (FRAM), the team identified inefficiencies in the Advanced Life Support (ALS) process, with delayed responses increasing patient mortality risks. Interviews with staff helped create "work-as-imagined" (WAI) and "work-as-done" (WAD) models, highlighting the underperformed functions like ward entry protocols, and fully stocked crash trolleys. Recommendations, including access cards, stock changes, and live simulations, were implemented, in an aim to improve ALS provision.

## Introduction

The aim of this Quality Improvement Project (QIP) was to find flaws in the current system in which clinicians respond to crash calls to Park House Mental Health Hospital due to past issues with access to the ward, equipment, and lack of experience of these events by the Mental Health team. Functional resonance analysis method (FRAM) modelling will be used to allow mapping of this system/process. From mapping this system, flaws will be noted, and therefore adaptations can be made in this process. Finally, these adaptations, once in place, will be subject to remodelling to see if this process is smoother and if any further improvements could be made.

Summary

The North Manchester General Hospital (NMGH) Crash Team covers Park House (Mental Health Hospital) for crash calls. These often do not go to plan and crashes within the NMGH building due to the aforementioned causes. Crash calls are a complex process, and thus an audit would not have been appropriate to assess these. FRAM modelling is an excellent way to map this complex system in order to fully understand the intricacies of this process as the FRAM allows us to deeply understand what is actually occurring and make positive changes.

FRAM

FRAM, created by Hollnagel in 2004 [1], is a way to look at complex systems holistically and has been used in many industries worldwide. It enables us to map a collection of functions (hexagons) and how these functions are linked together [1]. It also enables us to see what the system requires for these actions/functions to occur and thus enables the people involved to understand the limitations of the system. Even more importantly, in some situations, the FRAM enables us to see positive adaptations in processes performed by the staff members and thus allows us to recognize these actions and place these adaptations into protocols/pathways, encouraging more staff members to perform these tasks in such ways. This change in protocol would thus lead to a positive inflection in outcomes and reduce the variance between team members, meaning there should be a better standard of care for patients. The FRAM enables looking at processes in both a ‘Safety-I’ (the absence of things that are ‘safe’) and ‘Safety-II (the presence of things that make practice ‘safer’) - this is how the NHS, since 2015, has wanted us to critically analyse processes as defined in ‘From Safety-I to Safety-II: A White Paper’ [2].

A FRAM model is made up of hexagons. These hexagons have ‘functions’ within them and on their corners have six different variables that could affect this function: input, output, time, control, pre-condition, and resources.

The software called the FRAM Model Visualiser (FMV) allows the mapping of FRAMs to occur more easily. This is the software used for this QIP.

Cardiac arrests

Cardiac arrests have poor outcomes, with only 53% of in-hospital arrests achieving return of spontaneous circulation (ROSC) and 24% of patients recovering well enough to be discharged home [3]. There is also data to suggest that the slow response of the cardiac arrest team leads to negative outcomes. An example of this is that patient mortality reduces by 10% for every one minute that CPR / defibrillation is delayed, as defined by the Resuscitation Council UK [4]. The Resuscitation Council UK also states in its guidelines that defibrillation must occur within three minutes of recognition of cardiac arrest if clinically indicated [5]. To caveat this, if a cardiac arrest did occur at Park House, CPR and defibrillation should be occurring prior to crash team arrival if clinically indicated. Finally, also stated in the guideline is the phrase ‘Start ALS as early as possible’, and thus this QIP should improve this element of the Resus Council UK guideline [5].

## Materials and methods

Staff members who respond to cardiac arrest calls, which included both Park House doctors and those on the crash team at NMGH, were interviewed. The staff members were asked one question: ‘What do you think slows down/stops adequate ALS (Advanced Life Support) being performed when called to an arrest at Park House?’ via a Google Form (see Appendix). Further questions were asked if required from their initial Google Form response via a face-to-face interview in order to build the FRAM models. In total, over 30 crash team staff members were questioned, all of whom were doctors from the FY1 to ST7 levels, with 10 of these staff members further questioned via a face-to-face interview. 

From the answers of the systematic questioning of the healthcare teams, two FRAM models were created: a 'work-as-imagined’ (WAI - what we expect to happen) and a 'work-as-done’ (WAD - what actually occurs’). These FRAMs have been made by using the FRAM Model Visualiser (FMV) software.

## Results

Work-as-imagined (WAI)

**Figure 1:**
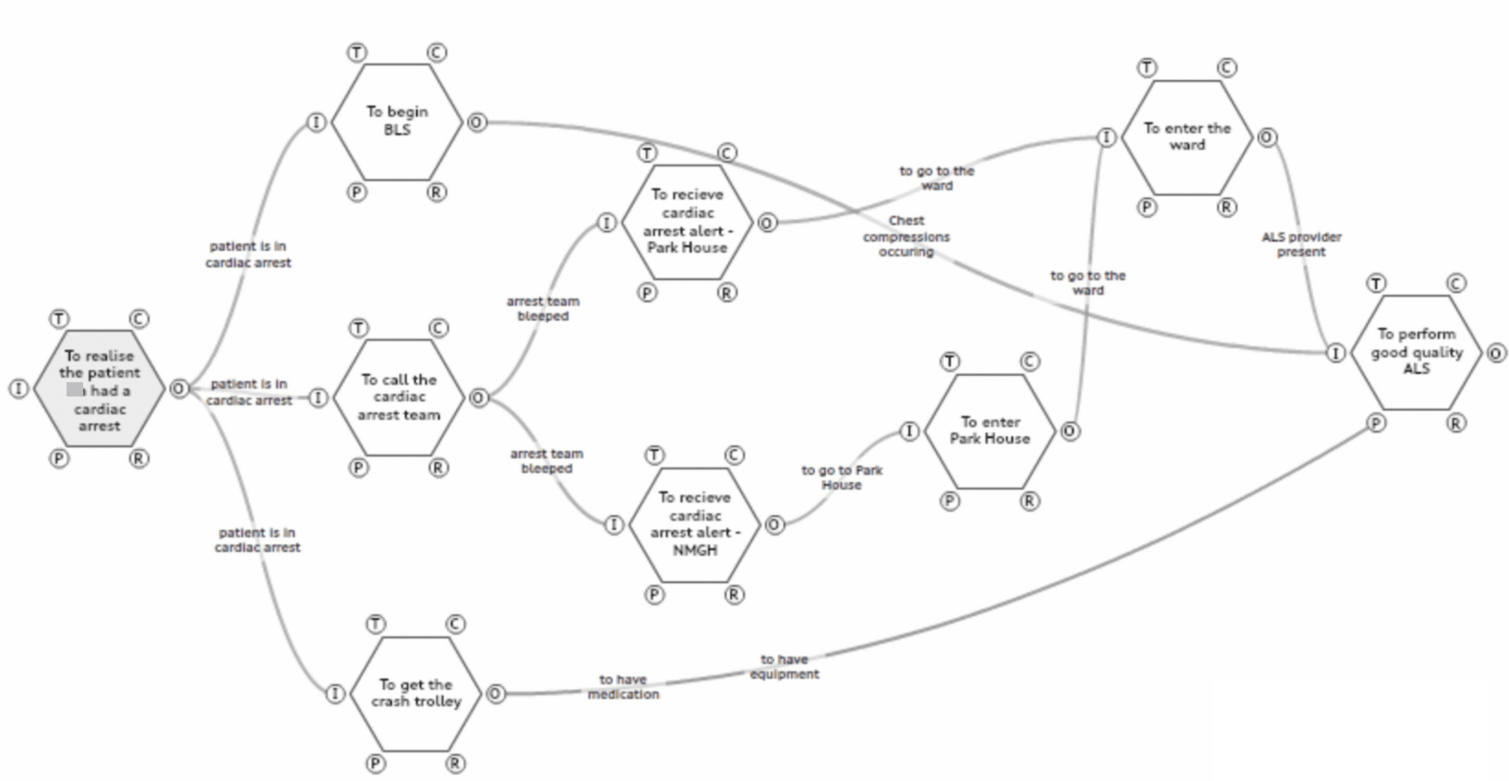
Work-as-imagined - a functional resonance analysis method (FRAM) model of what we expected the process to be like

This WAI FRAM model (Figure 1) represents what is believed as the process from the recognition of cardiac arrest to ALS occurring. Each hexagon represents a function, and the lines in between these functions represent how these functions are linked. Functions start in this map from the recognition of cardiac arrest and end at the performance of ALS. Within these two functions, several functions have to occur for the final function (performance of ALS) to occur.

Work-as-done (WAD)

**Figure 2:**
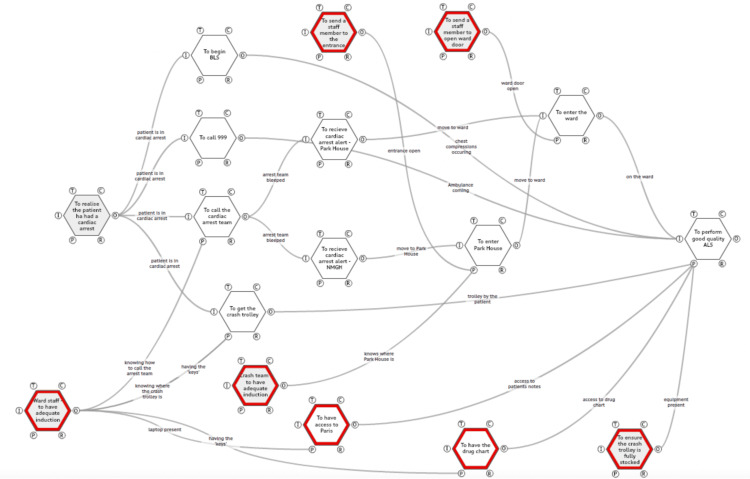
Work-as-done - a functional resonance analysis method (FRAM) model of what occurred during a cardiac arrest call at Park House

This WAD FRAM model (Figure 2) represents what actually occurs (if the process is done well) as the process from the recognition of cardiac arrest to ALS occurring with the functions in red required to make the process work effectively but often not done/forgotten, leading to these functions making the process weaker or slowing it down. Again, as with the WAI, the functions start in this map from the recognition of cardiac arrest and end at the performance of ALS. 

In this results section, the focus of the analysis will be on the red functions, key tasks that are either poorly performed or take too long to complete, as these will be the primary targets of the QIP.

One of the most critical areas for improvement is ensuring that ward staff, particularly registered nurses, receive an adequate induction. This is essential due to the significant responsibilities associated with their role. For instance, nursing and support staff must know how to correctly call the arrest team, explicitly stating both the ward name and ‘Park House’ to avoid confusion. Some doctors interviewed reported that only the ward name had been given in certain cases, leading to delays in the crash team reaching the correct location.

Another crucial aspect of induction is ensuring that staff have immediate access to keys. The crash trolley is stored in a locked clinical room for safety reasons, meaning that having the necessary keys on hand at all times is vital for accessing essential emergency equipment and medications. In addition, the drug charts, another red function in the FRAM model, are kept in this room, further reinforcing the importance of easy and timely access.

Induction must also include guidance on the use of laptops, which are required for reviewing patient notes and documentation, as there are no desktop computers available in the main ward area. As shown by the FRAM model, staff need to be aware that they must log into ‘Paris', the electronic record system used at Park House, a system to which the crash team does not have access.

Similarly, the crash team itself requires a thorough induction to ensure they are familiar with Park House, understand their responsibility for responding to crash calls there, and are aware of the correct entrance to use. Many staff members reported that when they first attended Park House, they lacked this crucial information, which hindered their ability to respond effectively to emergencies.

Another key issue identified is the need to send a staff member to the entrance when the crash team arrives. Since the external doors are often locked, and crash team members do not have key-card access, failure to do so results in delays that can postpone the initiation of Advanced Life Support (ALS), part of which is defibrillation. Defibrillation has been shown to increase mortality by 10% for each minute there is a delay [5]. Likewise, a staff member should also be designated to open the ward door itself, as the crash team has reported instances where they were left waiting outside, unable to pass through the ‘airlock’ to reach the ward.

Finally, ensuring that the crash trolley is consistently fully stocked remains a vital priority. Although regular checks are conducted at Park House, issues have still been reported with equipment stocks, suggesting that further measures may be required to guarantee the availability of all necessary equipment during an emergency.

In addition, some other points not related to the process itself were flagged as reasons ALS were delayed, yet these may not be changes that are as simple to make/factors that are fixed. These include the location of Park House (slower arrest team response); the inability to run urgent tests, such as blood gasses, easily; the nursing staff being psychiatric and not medically trained and thus less well versed with resus protocol; the Park House Doctors not being ALS trained, so for ALS to occur, the crash team are required; a 999 ambulance must be called promptly when the crash call is made as if ROSC (return of spontaneous circulation) occurs or the patient needs a transfer, and an emergency ambulance will be needed.

Simulation

Due to the findings of the initial two FRAM models, a simulation was organised in one the gardens, of a ward, at Park House. This was organised by members of the crash team, members of the senior leadership team, and members of the resuscitation team at both MFT and GMMH. The staff members at GMMH were not informed about the simulation prior to it occurring. The simulation site was chosen to be in a garden as this is an area that can be cordoned off from patients but still would test the problems found previously regarding access to the ward. Additionally, this would test the use of grab bags (mobile resuscitation kit) as this was something that did not come to focus in the first cycle of data collection as there had not been any recent emergency calls to areas without a resuscitation trolley.

Simulation findings

The simulation reinforced previous findings and provided further learning opportunities. It highlighted that the 2222 emergency call system was unclear regarding where staff members should be sent, leading to potential delays in response.

Access to both the building and the ward proved challenging, as no ward staff were assigned to assist the crash team in reaching the patient, further complicating the emergency response.

Another critical issue identified was that the grab bag lacked the essential equipment required for ALS. Notably, there was no adrenaline or amiodarone, both of which are time-critical medications, according to ALS algorithms [5].

There were also delays in accessing patient notes or finding a computer with the necessary records, which hindered efficient decision-making.

In addition, the crash team did not have access to a manual defibrillator, relying solely on an automated external defibrillator (AED). Capnography, a carbon dioxide trace used to aid the confirmation that an intubation is successful, was also unavailable, both of which made clinical decision-making more challenging during the cardiac arrest scenario.

A debrief was undertaken post-simulation, led by the MFT Resuscitation Lead, an emergency medicine consultant. 

**Figure 3:**
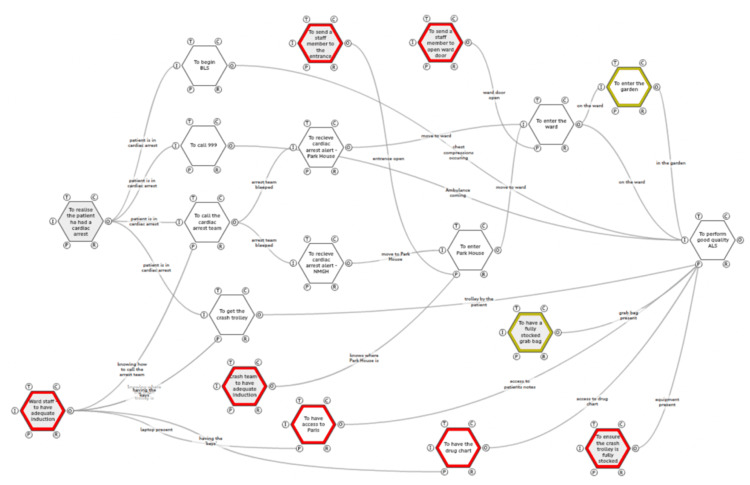
An updated work-as-done functional resonance analysis method (FRAM) model of what processes differed during the simulation in the garden at Park House

Below, Figure 3, is an extended FRAM model to include the findings from the simulation with the functions specific to the garden/simulation in green.

## Discussion

From the results of both the interviews and simulation, an accurate FRAM model was created of what occured during cardiac arrest calls to Park House which aided adaptations to be made to improve patient care. 

Adaptations made

Several adaptations were implemented as a result of this QIP and FRAM. A reminder was placed in the medical handover room to remind staff that they are responsible for providing ALS at Park House. There was also written information on how to get to Park House provided on the whiteboard in the handover room.

To improve access, Park House entry badges were issued to the medical registrars on the Crash Team, ensuring they could enter the building without delays. In addition, a script was placed on each phone within Park House, providing clear instructions on how to put out a cardiac arrest call and what information to include when doing so.

To facilitate smoother entry for the crash team, a reminder was placed on top of the crash trolley, instructing staff to send someone to the locked doors to allow the team access to both the hospital and the ward. ALS algorithms were also included in the grab bags to ensure staff had immediate access to essential guidance during resuscitation. Finally, pre-filled syringes of adrenaline and amiodarone were added to the grab bags, ensuring that these time-critical medications were readily available when needed. These were checked on a weekly basis. 

Results: post adaptations (second cycle)

Further interviews were conducted following the adaptations, targeting the same cohort of staff who had participated in the initial assessment before the changes were implemented. The purpose of this follow-up was to determine whether the adaptations had led to a significant improvement in the management of cardiac arrests at Park House. A total of 21 staff members, all of whom had responded to the initial questionnaire. This follow-up was done by repeating the initial questionnaire with an emphasis on the topics of access to wards, crash trolley stock, and 2222 calls.

One hundred percent of the staff members interviewed reported that having an access card had improved the time taken to provide ALS. Similarly, 100% of staff agreed that the inclusion of ALS algorithms in the grab bags had enhanced ALS provision by reducing variability in practice. There was also a 100% agreement that the availability of pre-filled syringes of adrenaline and amiodarone had reduced delays in administering these time-critical medications.

The addition of a reminder to send staff to the doors at Park House was seen as a positive change, with 95% of staff stating that it had improved access. This improvement may also have been influenced by the debrief sessions following simulations. In addition, 100% of the staff members confirmed that the introduction of the 2222 script had reduced variability in emergency calls, thereby hopefully minimising the risk of delays in initiating an effective cardiac arrest response.

Despite these improvements, there was a 100% agreement that further training and additional simulations are still required for Park House staff to continue enhancing ALS provision.

## Conclusions

Simple changes could greatly improve the factors that slow/prevent clinicians from performing ALS during crash calls to Park House. However, some factors are fixed or unrealistic to change. Both FRAM modelling and simulations are ways in which multiple limiting factors can be identified at one time within complex systems. This enables management to make multiple changes, often what is required to make a significant improvement within a complex system. All adaptations made, via identification from FRAM and simulations, are reported to have made a positive change in the provision of ALS at Park House.

## Figures and Tables

**Figure 4:**
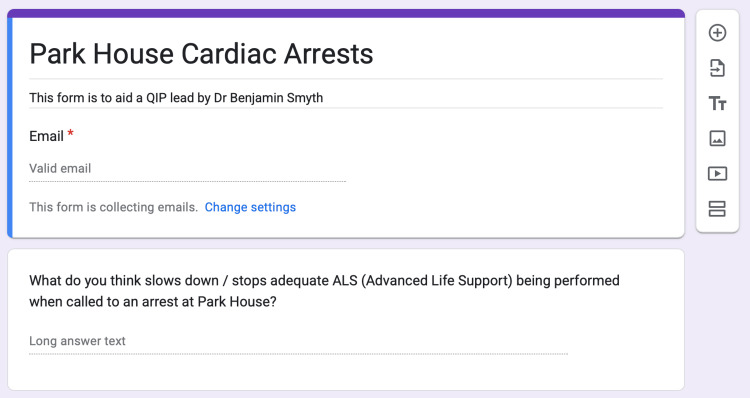
The questionnaire for data collection used to help build the work-as-imagined functional resonance analysis method (FRAM) model
